# Anti-nucleosome antibodies increase the risk of renal relapse in a prospective cohort of patients with clinically inactive systemic lupus erythematosus

**DOI:** 10.1038/s41598-020-69608-5

**Published:** 2020-07-29

**Authors:** Norma Alejandra Rodriguez-Jimenez, Edsaul Emilio Perez-Guerrero, Jorge Ivan Gamez-Nava, Dalia Isabel Sanchez-Mosco, Ana Miriam Saldaña-Cruz, Miriam Fabiola Alcaraz-Lopez, Nicte Selene Fajardo-Robledo, Jose Francisco Muñoz-Valle, David Bonilla-Lara, Valeria Diaz-Rizo, Laura Gonzalez-Lopez

**Affiliations:** 1 Departamento de Fisiología, Centro Universitario de Ciencias de La Salud (CUCS), Universidad de Guadalajara (U. de G.), Guadalajara, JAL Mexico; 2 Instituto de Investigación en Ciencias Biomédicas (IICB), CUCS, U. de G, Guadalajara, JAL Mexico; 3 Unidad Biomédica 02, Hospital de Especialidades, Centro Médico Nacional de Occidente (CMNO), Instituto Mexicano del Seguro Social (IMSS), Guadalajara, JAL Mexico; 4 Programa de Doctorado en Farmacología y Programa de Doctorado en Salud Publica, CUCS, U. de G, Sierra Mojada 950, 44340 Guadalajara, JAL Mexico; 5 Centro de Rehabilitación E Inclusión Infantil Teletón (CRIT) Guerrero, Acapulco de Juárez, Gro Mexico; 6 Departamento de Medicina Interna-Reumatología, Hospital General de Zona 45, IMSS, Guadalajara, JAL Mexico; 7 Laboratorio de Investigación Y Desarrollo Farmacéutico, CUCEI, U. de G, Guadalajara, JAL Mexico; 8 Departamento de Disciplinas Filosófico, Metodológicas E Instrumentales, CUCS, U. de G., Guadalajara, JAL Mexico; 9 Departamento de Medicina Interna-Reumatología, Hospital General Regional 110, IMSS, Guadalajara, JAL Mexico; 10 Present Address: Centro Universitario de Ciencias de la Salud, Universidad de Guadalajara, Sierra Mojada 950, Col. Independencia, C.P., 44340 Guadalajara, JAL Mexico

**Keywords:** Systemic lupus erythematosus, Lupus nephritis

## Abstract

An important goal in the management of systemic lupus erythematosus (SLE) is the prediction of relapses. This study assesses whether anti-nucleosome antibodies (anti-NCS) increase the risk of renal relapse in inactive SLE. A prospective cohort of 115 patients with inactive SLE (M-SLEDAI ≤ 2) were followed for 12 months to assess the development of relapse (increase of M-SLEDAI ≥ 4) and specific renal flare (renal SLEDAI ≥ 4). At baseline, we identified potential risk factors for relapse, including anti-NCS. At baseline, 18 (16%) of the 115 patients with inactive SLE were anti-NCS positive. At the 12-month follow-up, anti-NCS-positive patients had a higher incidence of renal relapse compared to anti-NCS-negative patients (38.9% vs 13.4%, respectively). In Cox regression analysis, after adjusting for age, disease duration, anti-dsDNA, and immunosuppressive drugs, the presence of anti-NCS positivity at baseline increased the risk of renal relapse (HR: 5.31, 95% CI 2.03–13.92). Nevertheless, there were no differences in the incidence of other relapses in anti-NCS-positive versus anti-NCS-negative. Our results indicate that in inactive SLE, anti-NCS determination can be useful for identifying patients with a higher risk of developing renal relapse. Interestingly, this study identified that continued use of oral immunosuppressive therapy in patients with inactive SLE can reduce the risk of renal relapse.

## Introduction

Systemic lupus erythematosus (SLE) is a chronic systemic autoimmune disease with different patterns of disease activity, including remission and episodes of relapse involving organs and tissues that produce a broad spectrum of clinical manifestations^1,2^. Several studies have identified a rate of relapse in SLE varying from 62 to 70% during one year of follow-up^3,4^. Hispanic individuals have an intermediate/high risk of relapse. Ugarte-Gil et al.^5^ observed that 56% of their cohort of Latin American patients experienced at least one relapse during disease evolution.

The kidneys are among the main organs involved in relapses. In SLE patients, the incidence of renal relapse varies from 27 to 67%^6^. Achieving a sustained remission of renal activity is a major goal of the treatment of lupus nephritis. Nevertheless, it has been estimated that approximately 38% of patients achieving renal remission have at least one major renal relapse within the next 5 years^7^. Additionally, the estimated cumulative probability of developing renal disease within 5 years of diagnosis in a Latin American cohort was 24% and 37% in SLE patients with or without the use of antimalarials, respectively^8^. One of the major problems faced by clinicians regarding relapse is that many renal relapses cannot be predicted using the current clinical biomarkers. Indeed, adequate clinical markers that are useful as predictors of renal relapse are required to adjust treatments and clinical care in patients with inactive SLE.

According to in vitro studies, the nucleosome is an important primary antigen in SLE^9^. Anti-nucleosome autoantibodies (anti-NCS) are part of a family of autoantibodies directed against chromatin, which have a higher affinity for intact nucleosomes^10^. Anti-NCS play a substantial role in the pathogenesis of certain organs involved in SLE, particularly in lupus nephritis. Li et al.^10^ identified in a cohort of 51 patients with SLE that anti-NCS positivity might be able to better discriminate between active and inactive SLE compared with other traditional biomarkers, including anti-dsDNA antibodies. Nevertheless, Li et al.^10^ also observed a low utility of anti-NCS antibodies to accurately predict clinical changes in these SLE patients. On the other hand, Ng et al.^11^ observed that anti-NCS levels might be influenced by the time since relapse^11^. Anti-NCS antibodies have also been associated with lupus nephritis^12^, and Manson et al.^12^ reported a decrease in anti-NCS levels when SLE patients were in renal remission. However, it is necessary to identify the value of anti-NCS in determining short-term clinical relapse and renal relapse in SLE patients with inactive disease. Furthermore, a multivariable approach excluding the effect of confounders it is still required to support the possible relation between anti-NCS antibodies and the development of renal relapse. To date, there are no longitudinal studies evaluating the role of anti-NCS as a predictive tool for the development of renal relapse in inactive SLE patients using multivariable approaches for controlling potential confounders. Therefore, we performed an observational prospective cohort study with the aim of identifying the utility of anti-NCS as risk factors for renal relapse in patients with inactive SLE using a multivariable Cox regression approach.

## Results

One hundred fifteen patients with inactive SLE were included in this prospective cohort study. Their general characteristics are described in Table 1. These patients had a mean age of 44.1 years, and 94% were female. At the time the cohort was formed, these patients had a mean disease duration of 10.5 years. At baseline, the mean M-SLEDAI was 0.60. At baseline, 16% of the patients were positive for anti-NCS, and 19% were anti-dsDNA positive. All the anti-NCS (+) patients were anti-dsDNA (+), and 4 SLE patients were anti-dsDNA (+) but anti-NCS (−) (kappa = 0.87). Furthermore, at baseline, 23 (20%) and 7 (6%) SLE patients had low complement C3 and C4 levels, respectively. These patients with inactive SLE have previous history of involvement to the following organs or tissues: mucocutaneous, 110 (96%); articular, 81 (70%); renal involvement, 55 (48%); blood involvement, 42 (37%); pericardial or pleural effusion, 11 (10%); and central nervous system involvement, 5 (4%). All the SLE inactive patients had history of anti-nuclear antibodies positive; and 35% had immunologic disorder (26.1% anti-dsDNA, 3.5% anti-Sm (+) and 6.1% anti-cardiolipin antibodies).

**Table 1:** General and clinical characteristics at baseline and rate of relapse in patients with inactive systemic lupus erythematosus included in the cohort.

	**n = 115**
**Demographics**	
Age (yr), mean ± SD	44.1 ± 11.5
Females, n (%)	108 (94)
**Comorbid diseases**	
Arterial hypertension, n (%)	28 (24)
Diabetes mellitus, n (%)	13 (11)
**Disease characteristics at baseline**	
Disease duration (years), mean ± SD	10.5 ± 12.2
M-SLEDAI score, mean ± SD	0.60 ± .0.92
Complement C3 low, n (%)	23 (20)
Complement C4 low, n (%)	7 (6)
Anti-NCS (+), n (%)	18 (16)
Anti-dsDNA (+), n (%)	22 (19)
**Drugs at baseline**	
Prednisone, n (%)	98 (85)
Immunosuppressive drugs (AZA or MMF) *	79 (69)
Chloroquine, n (%)	47 (41)
Methotrexate, n (%)	15 (13)
Prednisone doses, (mg/day), mean ± SD	9.4 ± 9.7
**Characteristics of relapse at follow-up**	
Development of any type of relapse, n (%)	46 (40)
Development of renal relapse, n (%)	20 (17)
**Severity of relapse**	
Mild or moderate, n (%)	39 (34)
Severe, n (%)	7 (6)
**Organs involved during relapse**	
Mucocutaneous, n (%)	66 (57)
Renal, n (%)	20 (17)
Musculoskeletal n (%)	19 (16)
Blood system, n (%)	3 (2.6)
Central nervous system, n (%)	3 (2.6)

Qualitative variables are expressed as percentages. Quantitative variables are expressed as the mean and standard deviation. M-SLEDAI: Modified Systemic Lupus Erythematosus Disease Activity Index. Anti-NCS: Anti-nucleosome autoantibodies. Anti-NCS (+) was considered when ≥ 14 RU/mL. AZA: Azathioprine. MMF: Mycophenolate mofetil. * Azathioprine 61 (53.0%); Mycophenolate mofetil 18 (15.6%). The blood system included leucopoenia (n = 3) and thrombocytopaenia (n = 2). The central nervous system included cranial nerve disorder (n = 1) and seizure (n = 2).

Current treatment of the patients with inactive SLE at time of the cohort assembly were: glucocorticoids 85%; oral immunosuppressive drugs [azathioprine (AZA) or mycophenolate mofetil (MMF)] 69%; antimalarial drugs (chloroquine) 41%. During the one-year follow-up, 40% of the SLE patients (n = 46) developed a relapse, and 20 (17%) developed a specific renal relapse. Of the 46 patients with a relapse, 39 (34%) had a mild or moderate relapse, and 7 (6%) had a severe relapse.

Of the total number of patients with SLE included in cohort (n = 115); 74 (64%) had ≥ 5 years of disease duration (data are not depicted in tables). At baseline, there were no differences in the frequency of positivity for anti-NCS between SLE patients with disease duration ≥ 5 years (anti-NCS positive = 16%) and those with < 5 years of disease duration (anti-NCS positive = 14%, *p* = 0.8). Patients receiving immunosuppressive therapy had a similar frequency of positivity for anti-NCS (17%) compared with those without immunosuppressive therapy (12%, *p* = 0.6). Previous to the assembly of the cohort of patients with inactive SLE, 55 patients had history of nephritis. Of these patients with history of previous nephritis 12 (21.8%) were anti-NCS (+); on the other side, 60 patients with SLE without history of nephritis, 6 were anti-NCS (+), *p* = 0.08).

Among 79 patients with inactive SLE at baseline treated with oral immunosuppressive drugs, 61 (77.2%) were treated with AZA and 18 (22.8%) with MMF. During the follow-up, MMF was substituted for AZA in two patients (3.2%), and six (9.8%) discontinued AZA (one patient after 3 months and five patients after 6 months of starting the cohort). All the discontinuations of AZA were attributed to an increase in transaminase levels. In addition, during the follow-up of the cohort, stable doses of immunosuppressive drugs were maintained in 43 SLE patients (70.5%) treated with AZA, whereas the AZA dose was decreased in 16 (26.2%). MMF was withdrawn during follow-up in six SLE patients (33%), all after 6 months of cohort assembly. The reasons for withdrawn MMF were an increase in transaminases in three patients (16.6%), and leukopenia in other three (16.6%). A stable dose of MMF was maintained in all the SLE patients who continued taking it during the follow-up.

Among the 115 SLE patients included in the study, prednisone doses were reduced in 41 patients (35.7%), and corticosteroid was withdrawn in 14 (12.2%), the rest (n = 60 patients with SLE) remained with stable doses. The mean baseline prednisone dose was 9.4 mg/day, and this mean dose decreased slightly to 7.3 mg/day during the follow-up, before the first relapse.

In Table 2, we show the comparison of variables between patients who developed renal relapse (n = 20) versus those who did not (n = 95). We observed a higher frequency of positivity for anti-NCS (*p* = 0.02) and a higher frequency of positivity for anti-dsDNA antibodies in patients with renal relapse (*p* = 0.02). We also found a lower frequency of immunosuppressive treatment in patients who developed renal relapse vs those with no renal relapse (50% vs 73%, respectively), though this trend did not show statistical significance (*p* = 0.06). Other variables did not achieve statistical significance.

**Table 2:** Comparison of baseline characteristics between patients who developed renal relapse during follow-up versus patients without renal relapse.

Variable	Renal relapse n = 20	Without renal relapse n = 95	*p*
Female, n (%)	17 (85)	91 (96)	0.10
Age (yr), mean ± SD	40.4 ± 12.2	44.9 ± 11.2	0.11
Disease duration (yr), mean ± SD	10.5 ± 7.1	10.5 ± 13.2	0.98
M-SLEDAI score at baseline, mean ± SD	0.7 ± 1.0	0.6 ± 0.9	0.59
Positive Anti-nucleosomes at baseline, n (%)	7 (35)	11 (12)	0.02
Positive Anti-dsDNA at baseline, n (%)	8 (40)	14 (15)	0.02
**Medication**			
Immunosuppressive drugs (AZA or MMF), n (%)	10 (50)	69 (73)	0.06
Prednisone doses, mg/day at baseline	10.0 ± 8.5	9.2 ± 9.9	0.74

Qualitative variables are expressed as frequencies (%). Quantitative variables are expressed as the means ± SD. Comparisons of qualitative variables were made with the χ^2^ test. Comparisons for quantitative variables were made with Student’s *t* test. M-SLEDAI: Modified Systemic Lupus Erythematosus Disease Activity Index; Anti-nucleosomes (+) were considered when ≥ 14 RU/mL. AZA: Azathioprine. MMF: Mycophenolate mofetil. Patients without renal relapse included those with no relapse or relapse other than renal relapse.

We also compared variables between SLE patients with any relapse independently of the organ involved (n = 46) versus those without any relapse (n = 69) (data are not depicted in tables). We observed a trend for a higher frequency of patients treated with oral immunosuppressive drugs among those who did not develop relapse (*p* = 0.06), whereas no differences were observed among the groups for disease duration, prednisone dose, or frequency of anti-dsDNA and anti-NCS positivity in SLE patients who developed relapses.

Nevertheless, patients with SLE anti-NCS (+), had a higher rate of more severe relapses (M-SLEDAI score > 8) (n = 3/18; 17%) compared with anti-NCS (−) SLE patients (n = 3/97; 4%, *p* = 0.05). Moreover, anti-NCS (+) SLE patients had a higher rate of specific renal relapse (39% vs 14%, respectively; *p* = 0.02). The level of anti-NCS was also increased in patients who developed renal relapses (median = 8.2 RU/mL) in comparison with patients who did not developed renal flares (median = 1.5 RU/mL, p = 0.05).

Table 3 shows the results of univariable analysis comparing the risk of developing any type of relapse and renal relapse at 6 and 12 months of follow-up according to the presence or absence of anti-NCS positivity. This table also shows the univariate relative risk for these types of relapse in the anti-NCS (+) group. For relapse in general, anti-NCS (+) at baseline was not associated with an increase in the risk of developing relapse. Instead, the presence of anti-NCS positivity increased the risk of developing renal relapse at 6 months (unadjusted RR: 3.85, 95% CI 1.37–10.79) and 12 months (unadjusted RR: 2.90, 95% CI 1.35–6.26) of follow-up.

**Table 3:** Risk of developing a renal relapse during the follow-up according to the positivity of anti-nucleosome antibodies at baseline. Univariate analysis.

Time of follow-up	Rates of relapse n, (%)	No relapse n (%)	RR	95% CI	p
**(a) Renal relapse**					
Follow-up: 6 months					
Anti-NCS (+) n = 18	5 (27.8)	13 (72.2)	3.85	1.37–10.79	0.02
Anti-NCS (−) n = 97	7 (7.2)	90 (92.8)			
Follow-up: 12 months					
Anti-NCS (+) n = 18	7 (38.9)	11 (61.1)	2.90	1.35–6.26	0.016
Anti-NCS (−) n = 97	13 (13.4)	84 (86.6)			
**(b) Any type of relapse**					
Follow-up: 6 months					
Anti-NCS (+) n = 18	4 (22.2)	14 (77.8)	1.08	0.42–2.78	1.00
Anti-NCS (−) n = 97	20 (20.6)	77 (79.4)			
Follow-up: 12 months					
Anti-NCS (+) n = 18	9 (50.0)	9 (50.0)	1.31	0.77–2.22	0.43
Anti-NCS (−) n = 97	37 (38.1)	60 (61.9)			

The incidence was computed according to the accumulative incidence. Anti-NCS (−): Negative anti-nucleosome antibody titres. Anti-NCS (+): Positive anti-nucleosome antibody titres. Anti-NCS (+) was considered when ≥ 14 RU/mL.

**Figure 1:**
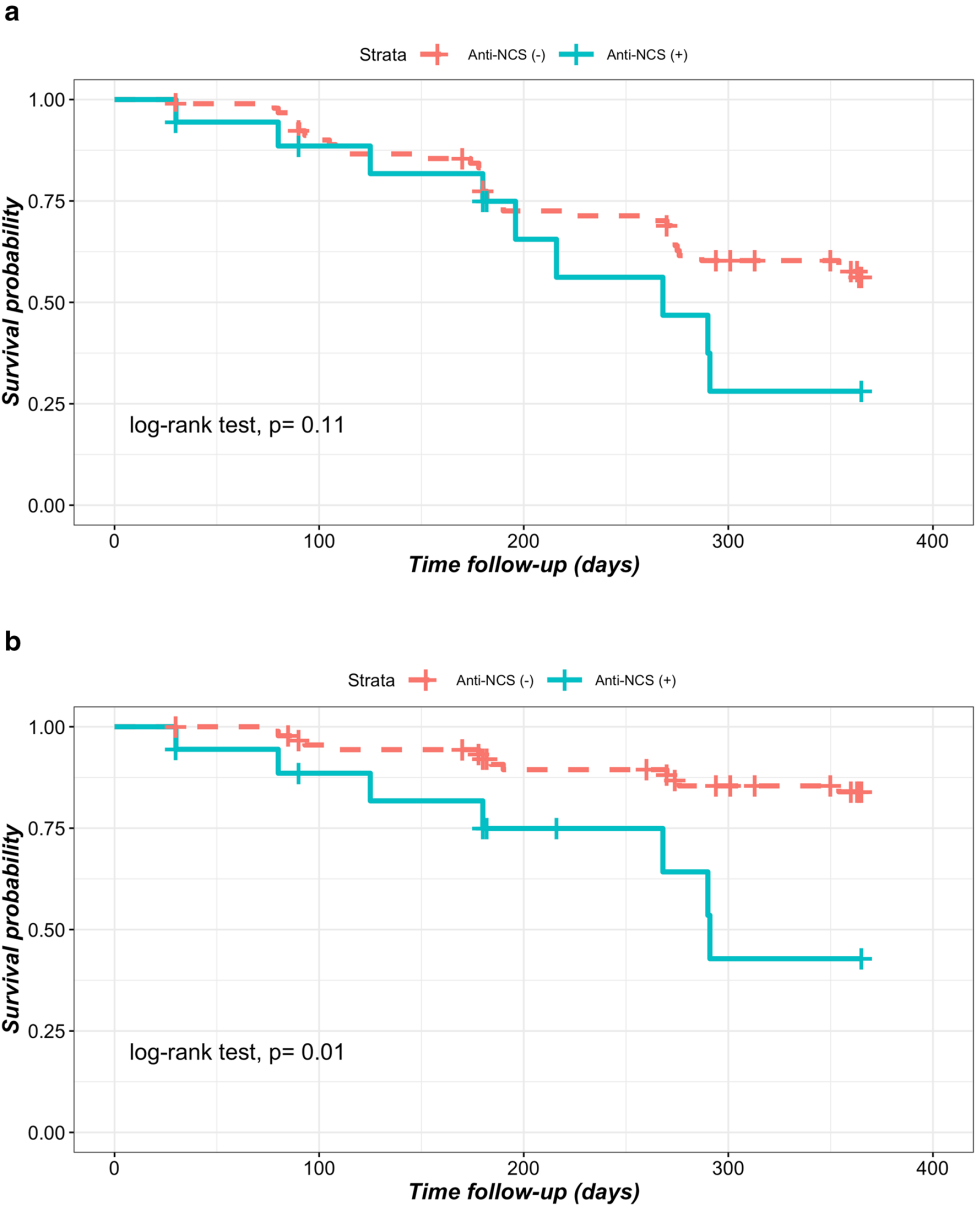
(**a**) Survival without any type of relapse. (**b**) Survival without renal relapse.

Figure 1 shows the Kaplan–Meier curves for the development of relapse (Fig. 1a) and development of renal relapse during the 12-month follow-up (Fig. 1b). In the graph continuous line (blue line) represents the survival curve of anti-NCS (+) group, and discontinuous line (red line) represents the survival curve of anti-NCS (-) group. As indicated in Fig. 1a SLE patients with anti-NCS (+) exhibited no differences in the incidence of relapse compared SLE patients with anti-NCS (−) (log-rank test, *p* = 0.11). On the other side, as shown in Fig. 1b, there was a higher rate of renal relapse in SLE patients with anti-NCS (+) group than in the SLE patients anti-NCS (−) group (log-rank test, *p* = 0.01).

Table 4 presents adjusted risk factors for the development of renal relapse and for the development of overall relapse using Cox regression analysis. After adjustment for age, disease duration, and anti-dsDNA positivity, the presence of anti-NCS (+), significantly increased the risk of renal relapse (HR: 5.31, 95% CI 2.03 to 13.92), whereas the persistence of treatment with oral immunosuppressive drugs (AZA or MMF) was a protective factor of renal relapse (HR: 0.28, 95% CI 0.12 to 0.70). Only persistence of treatment with oral immunosuppressive drugs (AZA or MMF) was a protective factor for the development of relapse (HR: 0.43, 95% CI 0.24 to 0.78), and no relationship with age, disease duration, anti-dsDNA positivity, or anti-NCS positivity was observed.

**Table 4:** Cox regression analysis of factors associated with relapse and with renal relapse.

Variable	Hazard ratio (95% CI)	*p*
**(a) Renal relapse**		
Positive Anti-nucleosomes at baseline	5.31 (2.03–13.92)	0.001
Immunosuppressive drugs (AZA or MMF)	0.28 (0.12–0.70)	0.006
Positive anti-dsDNA at baseline	Not in the model	
Age, years	Not in the model	
Disease duration, years	Not in the model	
**(b) Any type of relapse**		
Immunosuppressive drugs (AZA or MMF)	0.43 (0.24–0.78)	0.005
Positive anti-nucleosomes at baseline	Not in the model	
Positive anti-dsDNA at baseline	Not in the model	
Age, years	Not in the model	
Disease duration, years	Not in the model	

Cox regression analysis were performed using the stepwise method.

## Discussion

Our results showed that in SLE with inactive disease followed during one year around a 40% can develop at least one relapse, whereas almost 1 of 5 SLE patients with inactive disease are going to develop a renal relapse. A finding of anti-NCS positivity at baseline increased the 6-month risk for renal relapse more than threefold compared with that in SLE patients who were anti-NCS negative at baseline. In multivariate Cox regression analysis, in SLE clinically quiescent a finding of anti-NCS (+) assessed at the time of the cohort assembly significantly increased the risk of a future renal relapse, independently of potential confounders.

In SLE, several autoantibodies are utilized into the classification and diagnostic criteria; autoantibodies can be used as indicators of future disease outcomes, and some of the autoantibodies are markers of disease activity. Anti-dsDNA antibodies have been used by many authors as a possible marker of relapse in SLE. Ho et al.^13^, reported that a previous increment of anti-dsDNA titles increases the risk of relapse. Nevertheless, an important subgroup of patients with increased anti-dsDNA levels do not experience relapse. Therefore, the utility of these autoantibodies is still limited in a subgroup of patients.

Anti-NCS belong to the family of autoantibodies directed against histone epitopes exposed in chromatin. These autoantibodies are directed against conformational epitopes of the conjunction between dsDNA and core histones^14^. Anti-NCS have been considered useful for supporting the diagnosis of SLE^15,16^, obtaining similar or better results in terms of specificity than those obtained with anti-dsDNA^15,16^. A limitation of anti-NCS is the low sensitivity, including in SLE without immunosuppressive treatment. In a meta-analysis, Bizzaro et al*.*^17^, described a low sensitivity of approximately 59.9% of anti-NCS for SLE, though these autoantibodies displayed a high diagnostic specificity (94.9%).

Nevertheless, there is a lack of information about the frequency of anti-NCS in SLE patients with a long disease duration under immunosuppressive treatment and in patients with inactive disease. In our cohort primarily composed of SLE patients with a long disease duration, the frequency of anti-NCS positivity was 16%. The prevalence observed for anti-NCS was similar to that observed in studies performed by Amoura et al.^18^, and Lepers et al.^19^. Amoura et al.^18^, described a frequency of anti-NCS of 22% in SLE patients, whereas Lepers et al.^19^, found that 21% of their SLE patients were anti-NCS (+). Other cross-sectional studies have identified a higher prevalence of anti-NCS than that observed in our study, yet most of those studies assessed the prevalence of these autoantibodies in SLE patients with an active disease, with a high proportion having concurrent renal activity. In an elegant study, Simón et al.^20^^,^ observed a frequency of anti-NCS of 100%,however, the sample included SLE patients with severe disease activity. Shabana and colleagues also reported a prevalence of anti-NCS positivity reaching 92.1%, however, they described that these patients had at the time of the assessment a high prevalence of major organ involvement, including renal (37%), central nervous system (10.6%), lung (18.4%), and cardiac (5.3%) involvement and inclusive vasculitis (26.3%)^21^. In addition, Pradhan et al*.*^22^ reported a frequency of anti-NCS positivity of 80%, albeit among SLE patients with active disease. Therefore, most of the previous studies observing a high prevalence of anti-NCS included a high proportion of patients with active SLE. This seems to be a crucial aspect for understanding the differences between the low prevalence of anti-NCS observed in our study compared with previous works; indeed, only SLE patients with inactive disease were included in our cohort.

In this cohort, we identified that anti-NCS positivity at baseline increased the 6-month risk of renal relapse more than threefold compared with SLE patients who were anti-NCS negative, even though these autoantibodies were not predictors for overall relapse. These data in univariable analysis were consistent with the results of multivariable Cox regression analysis, in which positivity at baseline for anti-NCS remained an important predictor of renal relapse after adjusting for confounders.

Although anti-NCS have been associated with SLE activity, much of this information was derived from cross-sectional studies^23,24^. There are only a few cohort studies validating whether anti-NCS are useful as predictors of SLE relapse. Ng et al.^11^^,^ identified that the presence of anti-NCS positivity might predict lupus relapse in a retrospective cohort study of 27 SLE patients, supporting our findings. Regardless, that study had a retrospective design, which may be associated with limitations in reporting variables that might influence the results. Another relevant difference from our study is that the Ng cohort included only serologically active SLE patients with positivity for anti-dsDNA antibodies^11^. Instead, we included in our cohort patients with anti-dsDNA positive as well as patients with anti-dsDNA negative. On the other hand, Li et al.^10^ conducted a longitudinal study to evaluate whether anti-NCS might be useful as biomarkers in SLE. In a cross-sectional analysis, these authors found that anti-NCS have a sensitivity of 55%, specificity of 83% and positive predictive value of 96% as a biomarker of disease activity in SLE patients^10^. Although these authors found that anti-NCS correlate better than other traditional biomarkers in a longitudinal analysis, anti-NCS have poor utility for predicting subsequent changes in disease activity^10^.

Compared with previous studies, we used a different approach to assess the value of anti-NCS as a risk factor for renal relapse or relapse in inactive SLE. In a Cox regression analysis model, the hazard risk of anti-NCS positivity for the development of future renal relapse was 5.31. This was also an original study assessing whether the persistence of immunosuppressive treatment in inactive disease might act as a protective factor for the development of overall relapse. We observed that in patients with inactive SLE and persistent treatment with immunosuppressive therapy, the risk of subsequent renal relapse decreased by 72% compared with SLE patients with inactive disease who were not taking any immunosuppressive therapy. We consider that this information can be relevant in the clinical care to consider the persistence on immunosuppressive treatment in those SLE patients with inactive disease that present anti-NCS (+) or other risk factors of development a relapse. In SLE patients, relapses have been associated with a decrease in health-related quality of life^25^, high levels of chronic damage^26^ and an increase in mortality rates^27^. Furthermore, relapses constitute one of the main causes of hospitalization in SLE patients^28^. Therefore, further efforts must be made in order to detect the risk factors of future renal relapses.

The present study has some limitations that should be considered. First, because this cohort was designed with the aim of identifying the risk of relapse in patients with inactive SLE and anti-NCS (+) at baseline; we did not measure the anti-NCS titles during the follow-up. Even if these titles were no measured our study was able to identify that a finding of anti-NCS (+) in SLE inactive patients increases the risk of development a renal relapse. We also recognize in our study that the absence of anti-C1q antibody measurement is another limitation. C1q antibodies have been related with relapses and future studies should consider the measurement of these antibodies. A limitation inherent to prospective cohort studies is the possibility of changing the status of exposure in some patients. This was observed in a low proportion of our patients using oral immunosuppressive drugs or corticosteroids for whom medication was suspended before the end of the study. Nevertheless, these proportions of changing the exposure status were low, and therefore we do not consider that affected the validity of our results.

The present study has several strengths. First, the study design was a prospective cohort study. Therefore, we were able to identify a temporal relationship between anti-NCS (+) and the risk of developing renal relapse. This study was also able to show that the persistence of treatment with oral immunosuppressive drugs in SLE patients with inactive disease is a relevant protective factor, not only regarding a future overall flare but also the development of renal relapse. Another strength of this study was the inclusion of multivariable Cox regression analysis in the statistical analysis; using this strategy, we can be confident that our results demonstrate the independence of anti-NCS (+) and oral immunosuppressive drugs as factors associated with renal relapse. In Multivariable Cox regression analysis, variables potentially acting as confounders are excluded from the final model.

We conclude that although the prevalence of anti-NCS positivity is low in patients with inactive SLE, the presence of positivity for this autoantibody increases the risk of renal relapse at least threefold compared with that in inactive SLE patients negative for anti-NCS. Our results suggest that anti-NCS determination can be used as a marker to identify patients at risk for renal relapse, providing valuable information for clinical decision making. Based on these findings, we hypothesize that determination of the presence of anti-NCS antibodies in SLE patients in remission might be used to identify an anti-NCS (+) subgroup for whom oral immunosuppressive drugs should not be suspended due to an increased risk of renal flare in the next 12 months. Future controlled clinical trials comparing two arms, one of them continuing oral immunosuppressive drugs and another arm withdrawing oral immunosuppressive drugs in SLE patients with inactive disease and anti-NCS (+), are required to provide stronger evidence supporting this hypothesis.

## Methods

### Study design

Prospective cohort of SLE patients with inactive disease.

### Development study

For the inclusion phase, we identified SLE-inactive patients according to the Modified Systemic Lupus Erythematosus Disease Activity Index (M-SLEDAI)^13^ evaluated in a rheumatology outpatient clinic at a secondary-care centre in Guadalajara, Mexico (Department of Internal Medicine Rheumatology, Hospital General Regional 110 [HGR 110 del Instituto Mexicano del Seguro Social]).

We included patients who met the American College of Rheumatology (ACR) criteria for SLE^29^, diagnosed with SLE by a rheumatologist. Selection criteria: (a) ≥ 18 years old, (b) had at least 3 months of inactive disease as defined by M-SLEDAI scores from 0 to 2 points, (c) renal SLEDAI score of 0, (d) the duration of inactive disease was a minimum of 3 months and a maximum of 6 months and (e) were taking a stable dose of corticosteroid for at least 3 months before inclusion. Almost all SLE-inactive patients had a previous history of mucocutaneous involvement, approximately seventy percent had a history of articular involvement, almost a half had a history of renal involvement, and more than one-third history of blood involvement. Other organs, such as the pericardium, pleural or central nervous system, were involvement in a low frequency.

Exclusion: (i) patients with overlap syndrome, (ii) pregnant patients, and (iii) patients treated with cyclophosphamide, methylprednisolone, or rituximab. At the time of cohort assembly, a structured questionnaire, physical examination, and clinical chart assessment were performed by trained researchers. These researchers assessed age, disease duration, comorbid diseases including hypertension defined according to the guidelines of the American Society of Hypertension and the International Society of Hypertension^30^, and diabetes according to the American Diabetes Association^31^, history of organ involvement, time to the last relapse before study entry, and drugs used at the time of cohort assembly.

Laboratory tests included complete blood count, serum creatinine, urine analysis, creatinine clearance and 24-h urine protein. Anti-dsDNA antibodies were assessed by indirect immunofluorescence, and C3 and C4 were quantified by nephelometry.

### Characteristics of the cohort

All SLE inactive patients that were included in this study cohort were followed for one year or until the development of the first relapse unless they withdrew from the study. The SLE patients were scheduled to be assessed during the study follow-up at baseline, every 3 months for 12 months, or until the development of relapse. In addition, the patients were instructed to attend the clinic prior to their regular visit if they noticed any symptoms/signs suggestive of disease activity. At all visits, SLE patients were evaluated the same day by the researchers and rheumatologist to identify relapse with the modified SLEDAI (M-SLEDAI), described by Ho et al.^13^. M-SLEDAI is a validated disease activity index that removes the results of C3 and C4 complement fractions as well as, anti-dsDNA items that were included in the original SLEDAI described by Bombardier et al.^32^. The M-SLEDAI, therefore, contains 22 weighted items grouped into nine organ systems. Similar to the original SLEDAI, the total M-SLEDAI score is computed by summing all items that were present within the previous 10 days before the assessment. M-SLEDAI scores have a possible range from 0 to 101 points. High scoring reflex more severe disease activity. Researchers who assessed M-SLEDAI, were blinded to results of anti-dsDNA, C3, C4, and anti-NCS taken at baseline, until the end of study. During the follow-up at each visit, all patients were evaluated with M-SLEDAI and laboratory studies necessary to compute this index. A relapse of SLE was defined as an increase of ≥ 4 points on the M-SLEDAI during the follow-up with respect to the M-SLEDAI score obtained at baseline^33^. According to M-SLEDAI, a relapse can involve one or more organs. Mucocutaneus involvement was identified if there was any of the following findings: new maculopapular rash, alopecia, or mucosal ulcers. Relapses were classified arbitrarily into two levels: if the M-SLEDAI score increased 4–8 points (considered arbitrarily mild to moderate relapse) or if the relapse was an increase of > 8 points regarding the value of M-SLEDAI score at baseline (considered arbitrarily as a severe relapse). Renal relapse was considered using the parameters of renal SLEDAI^32^. A renal relapse was identified if patients presented at least one of the following manifestations: recent onset of proteinuria > 0.5 g/24 h, > 5 red blood cells/power field, > 5 white blood cells/power field, or granular or red-blood casts in urinalysis (excluding urolithiasis, urinary infections, menstruation or other causes).

Because this was an observational study, the therapy of the SLE patients was decided by the clinical rheumatologists in charge of the medical consult, modifying the type of drugs prescribed and doses according to their own criteria. This also included decisions of modifying doses, maintaining, or stopping medications, including corticosteroids and/or immunosuppressive drugs (azathioprine or mycophenolate). Two researchers assessed the clinical charts by extracting relevant information about the drugs prescribed at baseline and every SLE patient visit during the follow-up. This information included the prescription of oral immunosuppressive drugs (azathioprine and mycophenolate), corticosteroids and/or other drugs. The researchers collected all information about the dosage, permanence, maintenance or suspension of any of these drugs at baseline, and each visit during follow-up.

### Description of the aims of the prospective study

The study was designed to identify whether anti-NCS constitute a risk factor for the development of relapse. We compared two groups of patients with inactive SLE differing according to the presence or absence of anti-NCS at baseline. The patients who dropped out of the study for different causes were considered censored cases in the analysis, and person time of follow-up was computed. To preserve blinding of the researchers evaluating the patients with SLE, the anti-NCS results were not available until the end of the study.

### Anti-nucleosome antibody evaluation at baseline

Venous blood samples were taken from all patients with inactive SLE on the same day as inclusion into the cohort for identification of anti-NCS at baseline. The serum samples were coded and frozen at – 80 °C until the determination of autoantibodies. Quantification of anti-NCS was performed using a commercial ELISA kit (EUROIMMUN, Lübeck, Germany) according to the manufacturer’s instructions. All measurements for anti-NCS were performed by the same trained researcher, who was blinded to the clinical variables of the SLE patients. In our laboratory, the positivity value of anti-NCS was ≥ 14 RU/mL. As described above, the results of anti-NCS positivity or negativity were provided only at the end of the study to avoid expectancy bias and minimize the possibility of measurement bias.

### Statistical analysis

Quantitative variables are expressed as the means ± standard deviations (SD), and qualitative variables are expressed as frequencies and percentages (%). Proportions between groups were compared with the Chi-square test for those who developed relapse versus those without relapse, and Student’s t-tests were used to compare means between groups. A similar approach was used in sub-analysis between SLE patients who developed renal relapse versus those without renal relapse (this last approach included in the same group patients with relapse involving other organs and patients without relapse). We analysed the proportion of patients receiving oral immunosuppressive drugs who changed or stopped these drugs during the follow-up. Similarly, we analysed the proportion of patients for whom corticosteroids were suspended during the study and changes in the mean doses of corticosteroids during the follow-up. Because anti-NCS levels followed a non-normal distribution, we used the Mann–Whitney U test to compare the median levels of anti-NCS between patients who developed renal relapse vs patients with non-renal relapses. Kaplan–Meier survival analysis was employed to estimate the cumulative probability of developing relapse in SLE patients who were anti-NCS positive and negative. A similar approach was performed for renal relapse. We show survival graphs of these curves expressing the rate of relapse-free survival during the one-year follow-up. Differences in the survival time between exposed and unexposed groups were computed using the log-rank test. The relative risk of overall or renal relapse was calculated by comparing the incidence of relapse in the exposed group (SLE anti-NCS positive)/incidence of relapse in the unexposed group (SLE anti-NCS negative), at 6 and 12 months of follow-up; and 95% confidence intervals (95% CI) of the relative risks were calculated.

Cox proportional hazard models were used to identify variables associated with the development of any relapse and for specific renal relapses. Hazard ratios were computed to estimate risk for these relapses, including age, disease duration, positive anti-NCS and persistence of immunosuppressive drugs (azathioprine [AZA] or mycophenolate mofetil [MMF]) as covariates during the follow-up. The dependent variable was relapse for the first model, and for the second model the dependent variable was renal relapse which were adjusted for time until relapse. A multivariate Cox regression model was applied through the enter and stepwise methods. The probability of including and removing variables in the stepwise model was a *p* value of 0.05 or less and a *p* value of 0.10, respectively.

All analyses were performed using IBM SPSS ver. 23 statistical software (Statistics/IBM Armonk, NY, USA). Figures of survival curves were performed using *survival* and *survminer* packages for R version 4.0.0. The *p* value was set at a level of < 0.05.

### Ethics

The study protocol was revised and approved by Research and Ethics Board number 1303 of the Regional General Hospital of the Mexican Institute of Social Security (*Comité de Investigación en Salud del Hospital General Regional 110 del Instituto Mexicano del Seguro Social*), approval ID: R-2010-1303-15. This study was conducted following the guidelines of the Declaration of Helsinki. All participants signed informed consent prior to study onset.

## Data Availability

The datasets generated during and/or analysed during the current study are available from the corresponding author on reasonable request.
